# Air Should Not be There: A Case of Pneumomediastinum and Pneumopericardium in COVID-19

**DOI:** 10.7759/cureus.11696

**Published:** 2020-11-25

**Authors:** Shiqian Li, Edward Chau, Wesley Ghasem, Jina Sohn, Bassasm Yaghmour

**Affiliations:** 1 Pulmonary and Critical Care, University of Southern California Keck School of Medicine, Los Angeles, USA; 2 Internal Medicine, University of Southern California Keck School of Medicine, Los Angeles, USA; 3 Cardiology, University of Southern California Keck School of Medicine, Los Angeles, USA

**Keywords:** pneumopericardium, pneumomediastinum, covid-19, thoracic radiology, pulmonary critical care

## Abstract

Severe acute respiratory syndrome coronavirus 2 (SARS-CoV-2) is a novel coronavirus identified after widespread pneumonia cases in Wuhan, China at the end of 2019. This virus has been deemed a global pandemic and there remain many unknowns regarding the pathogenesis, management, treatment, and outcomes. This case report highlights a rare condition that possibly developed from the novel virus.

A 68-year-old Hispanic male with hypertension and gastroesophageal reflux disease, presented with two weeks history of fevers, chills, cough, and progressive shortness of breath. He was found to be positive for the novel SARS-CoV-2 upon admission. He rapidly developed severe acute respiratory distress syndrome (ARDS) secondary to his coronavirus disease 2019 (COVID-19) pneumonia requiring intubation and full ventilator support associated with acute anuric renal failure requiring emergent hemodialysis catheter placement and continuous renal replacement therapy (CRRT). Two weeks after being on mechanical ventilation and CRRT, he developed episodes of hypotension and tachycardia. A chest radiograph and computed tomography (CT) scan diagnosed pneumopericardium.

In the case presented, the patient’s CT of his thorax demonstrated bilateral ground-glass opacities and bilateral reticulations consistent with intraparenchymal injuries, most likely from his ARDS secondary to his initial SARS-CoV-2 infection. To date, there remains an unknown association between COVID-19 and causation of pneumomediastinum and pneumopericardium. There continues to be reports of clinically significant findings of pneumomediastinum and pneumopericardium in COVID-19 patients. It is known that COVID-19 causes dysregulated inflammation leading to diffuse alveolar damage and rupture, as well as myocarditis which may be the precipitant to the development of pneumomediastinum and pneumopericardium.

This case highlights the findings of pneumopericardium and pneumomediastinum in the novel SARS-CoV-2 virus. Given the multiple reported cases with similar time frames to the development of spontaneous pneumomediastinum in COVID-19 patients, an association between COVID-19 and spontaneous pneumomediastinum should be further studied.

## Introduction

Severe acute respiratory syndrome coronavirus 2 (SARS-CoV-2) is a novel coronavirus identified after widespread pneumonia cases in Wuhan, China at the end of 2019. This virus has been deemed a global pandemic and there remain many unknowns regarding the pathogenesis, management, treatment, and outcomes. At the time of writing, the virus has been shown to spread via airborne and droplet transmission. The most severe symptoms are when the infection spreads to the lower respiratory tract causing an acute respiratory distress syndrome (ARDS) like picture, leading to severe hypoxemia often requiring mechanical ventilator support. There have been many reported cardiac associations with coronavirus disease 2019 (COVID-19). Guo et al. demonstrated that myocardial injury is associated with fatal outcomes of COVID-19 and the inciting insult is thought to be due to dysregulated inflammation, putting patients at high risk for arrhythmia and ventricular dysfunction [1]. This dysregulated inflammation may serve as the nidus for both myocardial and pericardial injury and could potentially serve as a cause for pneumopericardium. Within the 2003 severe acute respiratory syndrome (SARS) coronavirus outbreak, there were documented cases of spontaneous pneumomediastinum unrelated to assisted ventilation, and more case reports of pneumomediastinum are being reported in association with COVID-19. This case highlights another typically rare finding of pneumopericardium and pneumomediastinum in the novel COVID-19 virus pandemic.

## Case presentation

A 68-year-old Hispanic male with hypertension and gastroesophageal reflux disease, presented with two weeks history of fevers, chills, cough, and progressive shortness of breath. He was found to be positive for the novel SARS-CoV-2 upon admission. He took no medications. He denied tobacco, alcohol, and illicit drugs. He rapidly developed severe ARDS requiring intubation and full ventilator support associated with acute anuric renal failure requiring emergent hemodialysis catheter placement and continuous renal replacement therapy (CRRT). Two weeks after being on mechanical ventilation and CRRT, he developed episodes of hypotension and tachycardia.

Physical exam findings

His baseline vital signs would be normotensive and sinus rhythm without any vasopressors, however, during these episodes of hypotension, his blood pressure would drop to 70/40 millimeters of mercury (mmHg), heart rate of 120 beats per minute (BMP), temperature of 36.4 degrees Celsius, and an oxygen saturation of 96%. Peak and plateau pressures on the ventilator were noted to be less than 30 cm H2O consistently. Physical examination revealed subcutaneous crepitus and bilateral basilar lung crackles. Cardiac examination was significant for water wheel heart sound, with regular rate and rhythm.

Diagnostic studies

**Figure 1:**
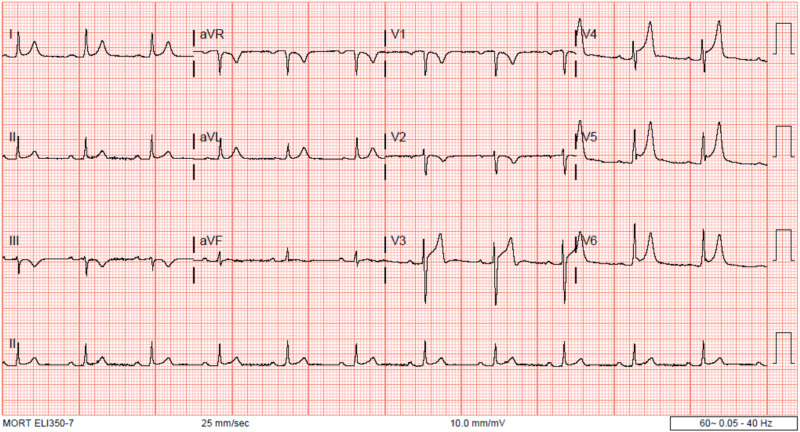
EKG showing ST elevations anteriorly.

**Figure 2:**
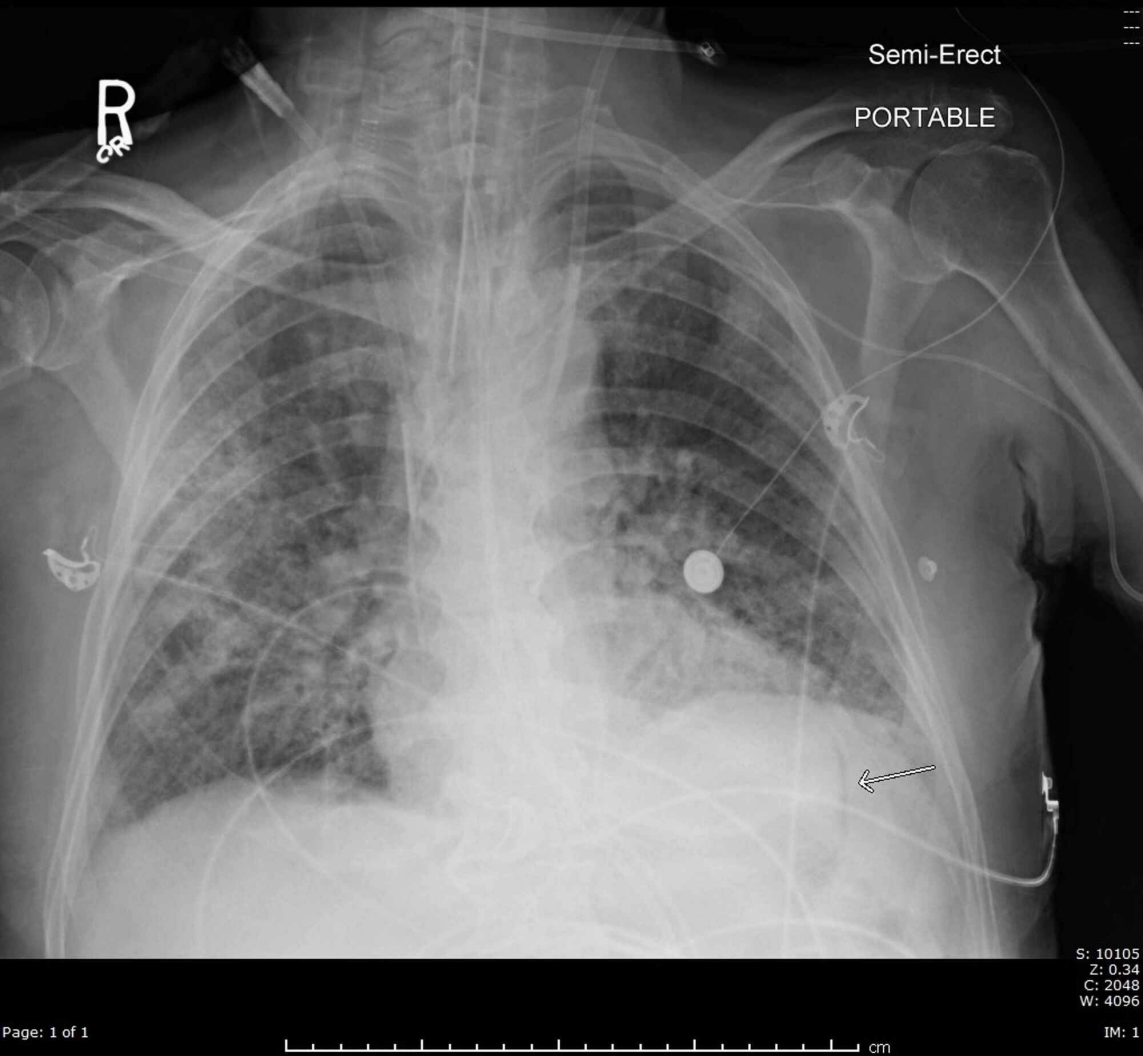
Chest X-ray showing pneumopericardium (arrow).

**Figure 3:**
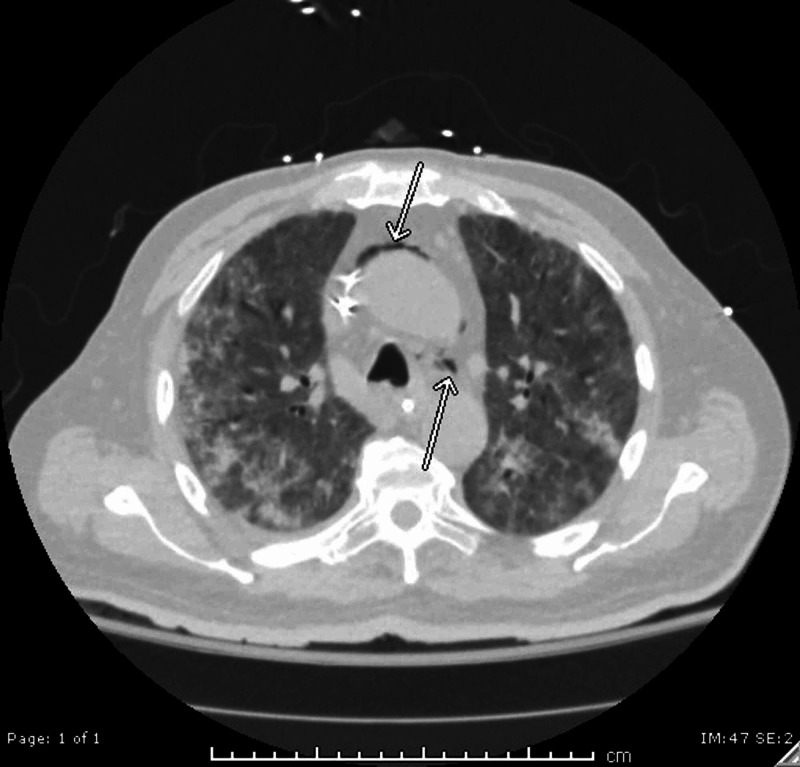
A lung window CT image of a superior portion of the thorax showing pneumomediastinum (arrows).

**Figure 4:**
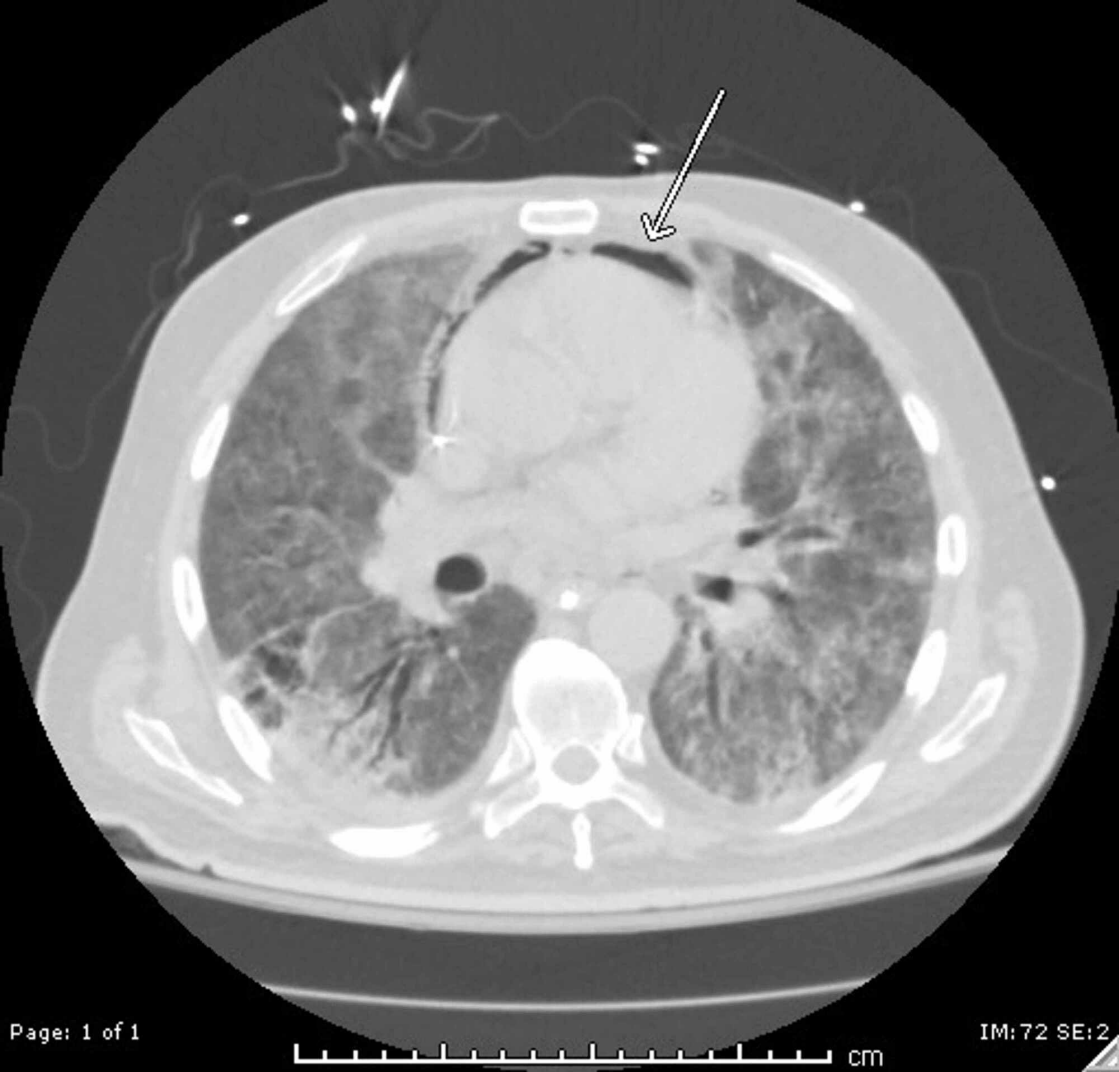
A lung window CT image of an inferior portion of the thorax showing pneumopericardium (arrow).

A stat electrocardiogram (EKG) demonstrated diffuse ST elevations greatest in the anterior segments (Figure 1). Transthoracic echocardiography demonstrated a hyperdynamic left ventricle with an estimated ejection fraction of >65%, a normal right ventricular size and function with no notable significant pericardial effusion as well as a minimal air gap during systole (Video 1). A crescent lucency overlying the left upper quadrant of the abdomen and extensive bilateral patchy opacification was seen on chest radiography (Figure 2). A chest computed tomography (CT) scan revealed air within the pericardial sac and within the mediastinum confirming the diagnoses of pneumomediastinum and pneumopericardium without pneumothorax (Figures 3-4). Blood work up was significant for leukocytosis, mild troponin-T elevation to 0.04 ng/mL. Blood cultures were negative. Respiratory culture revealed normal respiratory flora.

**Video 1:** Echocardiogram of a patient with COVID-19 with an air-gap sign concerning for pneumopericardium.

Clinical course conclusion

The patient would develop episodes of hypotension with blood pressures dropping to 70/40 mmHg range without any inciting event. This would occur for <30 minutes, happened infrequently, and generally while on renal replacement therapy. There were no arrhythmias or hypoxia during these events. Given the hyperdynamic ventricle noted on bedside ultrasound, the patient was resuscitated with intravenous albumin and his renal replacement therapy ultrafiltration was decreased. He was also given a short infusion of norepinephrine. Patient responded well to these interventions. Repeated EKGs showed resolution of ST-T wave changes and troponin was down trending. On subsequent chest X-ray and transthoracic echocardiography, there was no further evidence of pneumopericardium and improvement in the pneumomediastinum. The patient was eventually discharged to a long term acute care facility with tracheostomy and intermittent hemodialysis.

## Discussion

Pneumomediastinum, characterized as spontaneous (SPM) or secondary, is defined as air in the mediastinal space. Pneumomediastinum occurs when alveoli rupture allowing free air to dissect the bronchovascular sheaths towards and into the mediastinum. SPM has more favorable outcomes, however, definitive classification of spontaneous versus secondary continues to be discussed amongst experts. Pneumomediastinum symptoms include dyspnea and severe chest pain. On auscultation of the heart, “Hamman’s sign/crunch” can be heard, described as crackles synchronized with the beating of the heart. SPM is classified as a benign entity that will typically resolve on its own and with few episodes of recurrences in literature. A rare complication from pneumomediastinum is when a significant amount of air is entrapped in the mediastinum, defined as a malignant pneumomediastinum. This causes obstruction of the trachea and major vessels that are present in the mediastinum, which then would require decompression with thoracotomy. However, pneumomediastinum is most often treated conservatively with supportive measures and close monitoring with goals of symptoms relief.

Pneumopericardium is a rare condition defined as the presence of air or other gases within the pericardial sac. The first reported case of pneumopericardium was described in 1844 by Bricheteau as “bruit de Moulin” (water wheel sound) on physical examination associated with pneumopericardium [2]. This “water wheel sound” has also been described in air embolisms in the heart. In general, pneumopericardium is caused by one of four etiologies: trauma including blunt, penetrating, and barotrauma; fistulas between pericardial sac and air-containing organs (including the gastrointestinal system); secondary production of gas by bacteria inhabiting the pericardial space; or iatrogenic. In adults, most cases described are associated with procedural or traumatic injury to the pericardial space, while in children and neonates, most cases described are related to bronchial tree damage from mechanical injury in ventilator usage. Pneumopericardium and pneumomediastinum is often difficult to assess using echocardiography. Air is hyperechoic or white on imaging while fluid is hypoechoic or black on imagining. If there is large pneumopericardium, then echocardiogram will show no image of the heart. In most cases, there will be an absence of cardiac images during systole as the heart is pushed further away from the transducer by the air and then returns with diastole. This finding is also known as an “air gap sign” when seen using M-mode and is found in both pneumomediastinum and pneumopericardium [3]. One distinguishing factor between the two is the inability to see the heart in the subxiphoid view in pneumopericardium whereas in pneumomediastinum the heart is usually well visualized due to direct contact with the diaphragm without obstructing air artifact [4]. However, similar findings are often seen with respiratory interference such as tachypnea. There may be spontaneous bubbles or swirling bubbles in the pericardial space. In our case, the air in the pericardial space was small and this typical finding was not seen. Due to these issues with echocardiography, chest CT is the preferred method for diagnosis. 

To date, there remains an unknown association between COVID-19 and causation of pneumomediastinum or pneumopericardium. In a previous paper from the 2003 SARS coronavirus outbreak, there were documented cases of SPM unrelated to assisted ventilation. Chu et al. studied 112 cases and found 13 patients with SPM unrelated to ventilation with a mean time of symptom onset of 19.6±4.6 days [5]. They reasoned that the development of pneumomediastinum in the 2003 SARS coronavirus followed alveolar damage leading to interstitial emphysema that then dissected the bronchovascular sheaths into the mediastinum.

There have been now multiple documented cases of spontaneous pneumomediastinum in patients with COVID-19 including a case of a non-mechanically ventilated patient, however, the pathogenesis remains hypothesized [6-7]. When the patient of this case report developed pneumomediastinum and pneumopericardium, there was no literature citing an association to COVID-19. However, as the pandemic continued and more cases of COVID-19 developed, this case report's authors noted a significant increase in the literature of similar spontaneous pneumothoraces in patients with COVID-19. Similar to other case reports, the authors of this case report presume that the development of pneumomediastinum was spontaneous given no inciting events. It is known that COVID-19 causes dysregulated inflammation leading to diffuse alveolar damage and rupture similar to what was seen in the 2003 SARS coronavirus outbreak. This leads authors of this case report to hypothesize a similar pathophysiology occuring in the development of pneumothorax in COVID-19 patients. There are also case reports of COVID-19 causing myocarditis and pericarditis [8-9]. These injuries again appear to be caused by dysregulated inflammation. The authors of this case report hypothesize that this dysregulated inflammation acts as the nidus for myocarditis and pericarditis and can potentially sever the barrier between the pericardium to the mediastinum and if a pneumomediastinum is present, there would be a communication for the air to develop. Given the multiple reported cases with similar time frames to the development of SPM in COVID-19 patients, an association between COVID-19 and SPM should be further studied.

## Conclusions

This case demonstrates a rare finding in a novel coronavirus infection. The patient developed symptoms of shock and prompted a thorough evaluation that demonstrated the unexpected findings of pneumomediastinum and pneumopericardium. More studies are required to evaluate possible causation of pneumomediastinum and pneumopericardium secondary to COVID-19.
